# Revealing Atomic-Level Mechanisms of Protein Allostery with Molecular Dynamics Simulations

**DOI:** 10.1371/journal.pcbi.1004746

**Published:** 2016-06-10

**Authors:** Samuel Hertig, Naomi R. Latorraca, Ron O. Dror

**Affiliations:** 1 Department of Computer Science, Stanford University, Stanford, California, United States of America; 2 Biophysics Program, Stanford University, Stanford, California, United States of America; 3 Department of Molecular and Cellular Physiology, Stanford University, Stanford, California, United States of America; 4 Institute for Computational and Mathematical Engineering, Stanford University, Stanford, California, United States of America; University of North Texas System College of Pharmacy, UNITED STATES

## Abstract

Molecular dynamics (MD) simulations have become a powerful and popular method for the study of protein allostery, the widespread phenomenon in which a stimulus at one site on a protein influences the properties of another site on the protein. By capturing the motions of a protein’s constituent atoms, simulations can enable the discovery of allosteric binding sites and the determination of the mechanistic basis for allostery. These results can provide a foundation for applications including rational drug design and protein engineering. Here, we provide an introduction to the investigation of protein allostery using molecular dynamics simulation. We emphasize the importance of designing simulations that include appropriate perturbations to the molecular system, such as the addition or removal of ligands or the application of mechanical force. We also demonstrate how the bidirectional nature of allostery—the fact that the two sites involved influence one another in a symmetrical manner—can facilitate such investigations. Through a series of case studies, we illustrate how these concepts have been used to reveal the structural basis for allostery in several proteins and protein complexes of biological and pharmaceutical interest.

## Introduction

Allostery—the process by which biological macromolecules transmit the effect of a stimulus (typically ligand binding) at one site to a physically distinct site—is among the most important and heavily studied properties of proteins [1,2]. Many proteins require allostery to carry out their basic functions; for example, the binding of a hormone to a receptor may cause a structural change in that receptor, allowing the receptor to bind another protein and trigger cellular signaling. Other proteins rely on allostery for regulation; for example, small molecules that bind an enzyme at sites far from the catalytic active site often tune the enzyme’s activity. Identifying the atomic-level mechanisms of allostery is critical not only for understanding protein function but also as a foundation for structure-based drug design and protein engineering [3,4].

Determining the structural basis for allostery, however, often proves challenging. Allostery generally involves structural changes that are caused by a stimulus at one site and that also affect another site. These changes may be very subtle, and their investigation is complicated by the fact that even in the absence of a stimulus, proteins fluctuate constantly between ensembles of related conformations; a stimulus generally induces allostery by shifting this ensemble [5–7]. Such effects are often not evident from the static snapshots provided by crystallographic protein structures.

Molecular dynamics (MD) simulations have emerged as a valuable complement to experimental methods [8] in the study of allostery because they capture the motion of proteins in full atomic detail. These simulations—whose initial development several decades ago was among the achievements recognized by the 2013 Nobel Prize in Chemistry—predict the position of every atom in a biomolecular system as a function of time using Newtonian mechanics [9]. They have become substantially more powerful in recent years, thanks to improvements in computer power, improved simulation algorithms, and improved potential energy functions (known as force fields) that better approximate the underlying physics [10].

MD simulations have proven particularly useful in the investigation of allostery for two reasons. First, they provide an all-atom description of protein behavior with very high resolution in both space (sub-angstrom) and time (femtoseconds). Second, they allow a researcher to introduce carefully controlled perturbations to the biomolecular system—for example, through the addition of ligands or the application of external forces to localized regions of the protein—and to determine their effect on the protein’s structure and motion.

Here, we demonstrate how to use MD to study allostery. Several reviews have surveyed specialized simulation techniques designed to probe allosteric systems [11–13]. Our focus is instead on the fundamental concepts that guide the design of a simulation-based investigation of allostery in a biomolecular system. We hope that this article will prove useful both to structural biologists beginning to use MD and to MD practitioners turning to the study of allostery.

## Determining Binding Modes of Allosteric Ligands

Our primary focus in this review is on using MD to investigate the nature of allosteric coupling within a protein or protein complex (next section). MD has also proven useful, however, in determining the binding sites and poses of ligands previously shown pharmacologically to bind a given protein and in determining putative binding sites for which new ligands might be designed. In particular, these techniques can help determine binding sites for allosteric ligands and thus contribute to the study of allostery.

Thanks to the availability of faster and cheaper computing hardware, it has recently become practical to perform MD simulations in which ligands bind spontaneously to proteins without any prior knowledge of the binding site [14–16]. The most conceptually straightforward approach is to simply perform a long simulation in which one or more ligands are positioned randomly in the water surrounding the receptor. The ligands then diffuse around the entire protein before “discovering” their binding site. Such methods have been used to uncover binding sites and poses for allosteric ligands ([17] and Case Study 1). They may capture ligand binding events even when the protein needs to change conformation locally to allow binding.

The binding of certain ligands, however, takes place on timescales too slow to be captured by simulation, even on supercomputers. In some cases, one can circumvent this problem by using large numbers of short simulations together with statistical modeling techniques that describe long-timescale events as sequences of short-timescale events [14,18]. Alternatively, if the binding site is known, one can position the ligand within the binding site at the beginning of the simulation, allowing its pose and the local protein conformation to adapt during the simulation [19–21].

MD can also be used to find pockets on a protein that might represent targets for ligand design. In particular, some drugs bind to “cryptic” pockets that are not visible in a ligand-free crystal structure [22]. Such pockets may form during simulation, particularly if drug-like ligands are included in the simulation to help induce pocket formation [16,23–26]. These techniques may be used to discover binding sites different from the native binding site. Such alternative sites are often referred to as “allosteric,” sometimes even in cases when binding of ligands at these alternative sites does not have an allosteric effect on the native binding site.

## Determining the Nature of Allosteric Coupling

A more widespread application of MD simulations involves determining whether, why, and how a stimulus at one site on a protein or protein complex causes effects at other sites. In many cases, two particular sites have been shown experimentally to be allosterically coupled—for example, binding of a ligand at one site increases or decreases affinity or activity at another site—and one may wish to explain the molecular mechanism underlying this coupling, thus enabling the design of additional ligands with similar or different effects. In other cases, the goal is to discover what other locations on a protein might be allosterically coupled to a particular site of interest. Despite the diversity of allosteric proteins and allosteric effects, several common principles guide their investigation by MD simulations.

### Observing correlated motions

The most straightforward—and perhaps the most common—approach to studying allostery with MD simulations is simply to initiate a simulation from an experimental protein structure and then look for interdependent motions of residues at and between the sites of interest. These simulations are most often analyzed by visual inspection (using molecular rendering software such as VMD [27]) and calculation of simple measurements such as distances between pairs of atoms or dihedral angles of amino acid side chains.

Because MD simulations produce large amounts of data, and because interdependent motions can be subtle, these coupled motions can be difficult to pick out by eye. Several analysis methods have been developed specifically to find residues that are coupled allosterically (reviewed in [11,13]), using metrics such as mutual information [28,29], interaction energies [30], or correlated motion [31–34]. General MD analysis methods that automatically detect structural changes or identify structural substates may also be useful for identifying interdependent motions of interest [35–37].

Researchers sometimes speak of allosteric “pathways,” suggesting a picture in which a single continuous chain of residues between the two sites accounts for allosteric coupling. In reality, though, allostery between two sites is often mediated by difficult-to-define networks involving many loosely coupled residues or by global conformational changes involving the entire protein [38,39]. These different possibilities should be kept in mind when examining simulation results with an eye toward deciphering an allosteric mechanism.

### Introducing in silico perturbations

In some cases, one can identify allostery and explain its mechanism based simply on an “equilibrium” simulation—a simulation initiated from an experimental structure, with the same bound ligands as in the experimental structure and with no external forces applied. To characterize allostery in the physical world, however, one generally needs to apply a stimulus, such as adding a ligand, and the same is often true in simulation.

Indeed, one of the strengths of MD simulation for the study of allostery is that one can readily introduce a wide variety of well-defined perturbations to a biomolecular system in silico. Most importantly, one can add or remove ligands. Removing a ligand present in the experimental structure is particularly straightforward; adding a ligand that was absent in the experimental structure is more involved and requires knowledge of the ligand binding mode, but often this can be determined by MD (see previous section) or other computational or experimental methods. Other naturally occurring stimuli have easily implemented computational equivalents. For example, one can apply a mechanical force ([40] and Case Study 4) or transmembrane voltage [41], or one can modify protein protonation states to capture the effects of local changes in acidity [42].

The introduction of “artificial” perturbations in silico may also help to identify allosteric mechanisms. For example, one might force a site or residue in a protein into a particular conformation using techniques known as steered MD ([43] and Case Study 4), biased MD [44], or targeted MD [45] in order to see how a different site or residue responds. A number of specialized methods have been developed to characterize allostery by introducing small local alterations in position or motion and then scanning for resulting changes in the behavior of neighboring regions of the protein (for example, the perturbation-response–scanning method [46,47] or pump-probe MD [48]). Several other reviews provide more detail on such methods [11,13].

Finally, mutation of residues is straightforward to implement in silico. Mutagenesis differs from the other perturbations discussed above in that it involves changing the internals of the protein under study rather than perturbing it in the way that a change in its external environment would. Connecting mutagenesis results to the allosteric mechanism of the wild-type protein is not always straightforward. Mutagenesis is a widely used experimental technique, however, and replicating or predicting the results computationally can be of value.

Comparing simulation results across multiple conditions, including perturbed and unperturbed systems, requires careful analysis. One must first identify differences between simulations under different conditions and then determine the statistical significance of those differences. Quantitative analyses, including statistical tests, are often required to identify changes that result specifically from the application of a perturbation [49].

Deciding which perturbations to make, and to which starting structures, is perhaps the most critical aspect of designing an MD-based study of allostery. We reflect on this point in the following subsection and in the case studies.

### Bidirectionality and the principle of microscopic reversibility

**Fig 1:**
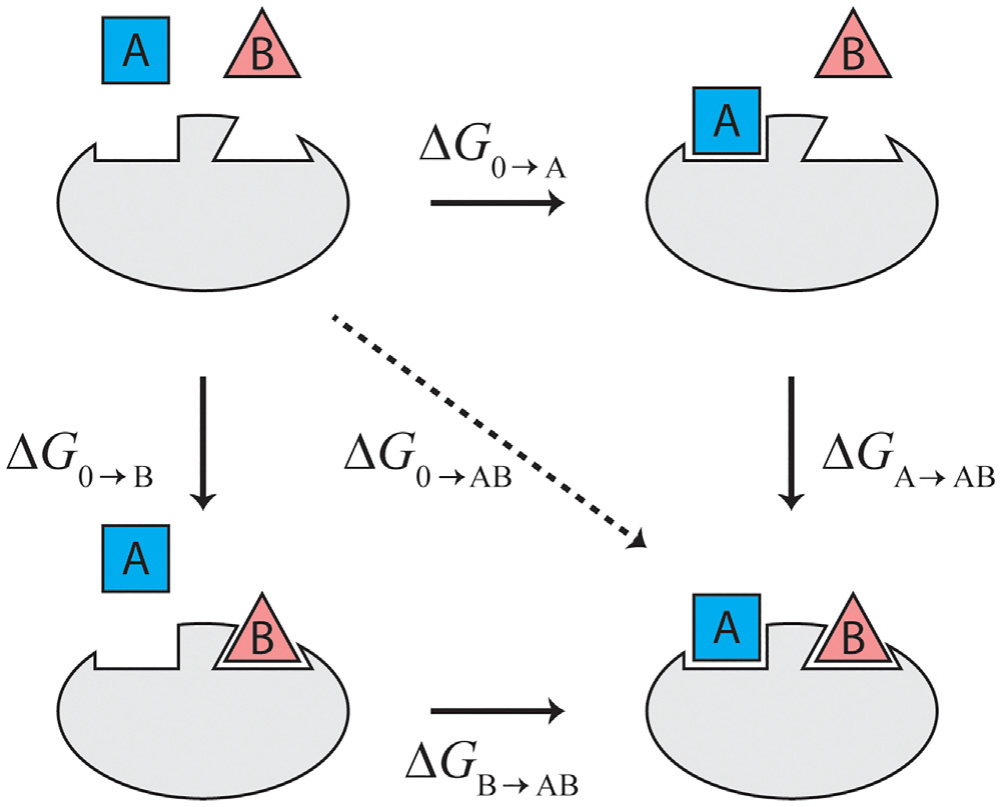
Bidirectional symmetry in allosteric systems. Suppose that a protein can bind two different ligands, A and B, each at their respective sites. The difference in free energy Δ*G*_0→AB_ between a state with both ligands bound and one with neither ligand bound is independent of the order in which the ligands bind, so we can write it both as Δ*G*_0→AB_ = Δ*G*_0→B_ + Δ*G*_B→AB_ and as Δ*G*_0→AB_ = Δ*G*_0→A_ + Δ*G*_A→AB_, implying that Δ*G*_0→B_ + Δ*G*_B→AB_ = Δ*G*_0→A_ + Δ*G*_A→AB_. Rearranging this equation yields Δ*G*_A→AB_ − Δ*G*_0→B_ = Δ*G*_B→AB_ − Δ*G*_0→A_. That is, the difference in the binding energy of ligand A with or without ligand B bound is the same as the difference in the binding energy of ligand B with or without ligand A bound. If one side of this equation is negative, the other must also be negative, and if one side is positive, the other must also be positive. Thus, if binding of ligand B is more favorable in the presence of A, then binding of ligand A is more favorable in the presence of ligand B (positive cooperativity). Likewise, if the presence of ligand A disfavors the binding of ligand B, then the presence of ligand B disfavors the binding of ligand A (negative cooperativity).

Two physical principles are particularly useful when designing MD studies of allostery. The first is the bidirectional nature of allostery—the fact that the effects of two sites on one another are symmetrical (Fig 1). For example, if binding of ligand A at site A increases the binding affinity of ligand B at site B (a situation known as “positive cooperativity”), then the binding of ligand B at site B will increase the binding affinity of ligand A at site A. (In fact, the two affinity increases will be exactly equal, if measured as changes in the free energy of binding.) This means that if one wishes to understand the allosteric effect of site A on site B, one could instead study the effect of site B on site A. Depending on the locations of the sites and the nature of the ligands involved, the latter simulations might prove much more feasible; for example, the required simulations might be much shorter (see Case Studies 1 and 3).

The second principle is microscopic reversibility, which states that, at equilibrium, a chemical system (e.g., a protein) that transitions between two structural states will follow exactly the same pathways in the forward and reverse directions [50,51]. This means that if we have two different experimental structures for a protein, we can choose to simulate the transition between them in either direction. Although the forward and reverse transitions will follow the same pathways (in the absence of an external energy input), one of them might occur much more quickly and thus be more amenable to simulation (see Case Study 2). Likewise, we can simulate a ligand-binding process to learn about the unbinding process, or vice versa [15]. Properly exploiting microscopic reversibility does require careful consideration of simulation initial conditions, although transitions initiated from well-defined physical states will generally be less sensitive to these initial conditions (for a thorough discussion, see [52]).

## Limitations of MD Simulations in the Context of Allostery

Both the design of MD simulations and the interpretation of their results should take into account the limitations of these simulations, several of which we highlight here. First, the force fields used to compute the forces acting on each atom during an MD simulation represent approximations to the underlying quantum mechanics. These force fields have improved substantially in recent years, but they remain imperfect [53]. Some types of simulation results are more likely than others to be affected by force field error, as discussed elsewhere [54]; such uncertainty should be taken into account in analyzing simulation results, and these results should be interpreted in the context of all available sources of information, particularly relevant experimental data.

Second, important biomolecular processes, including ligand binding and conformational change, sometimes take place on timescales longer than the timescales accessible by simulation, which range from nanoseconds to milliseconds depending on the simulation software, methodology, and hardware employed [10,55,56]. As we show in the case studies below, careful study design can help overcome these limitations in some cases.

Third, an accurate simulation typically depends on the availability of an accurate experimental protein structure, or a good homology model, for use as an initial condition. Design of simulation studies is thus heavily influenced by the availability of experimental structures.

## Case Studies

In order to illustrate the concepts described in the previous sections, we next present several case studies that used MD simulations to investigate protein allostery. We have chosen examples from our own previous work simply because we are most familiar with the details of these studies. In each example, we describe the rationale underlying simulation design, with a focus on the types of questions asked and the perturbations involved.

### Case Study 1: Structural basis for allosteric modulation of a GPCR

**Fig 2:**
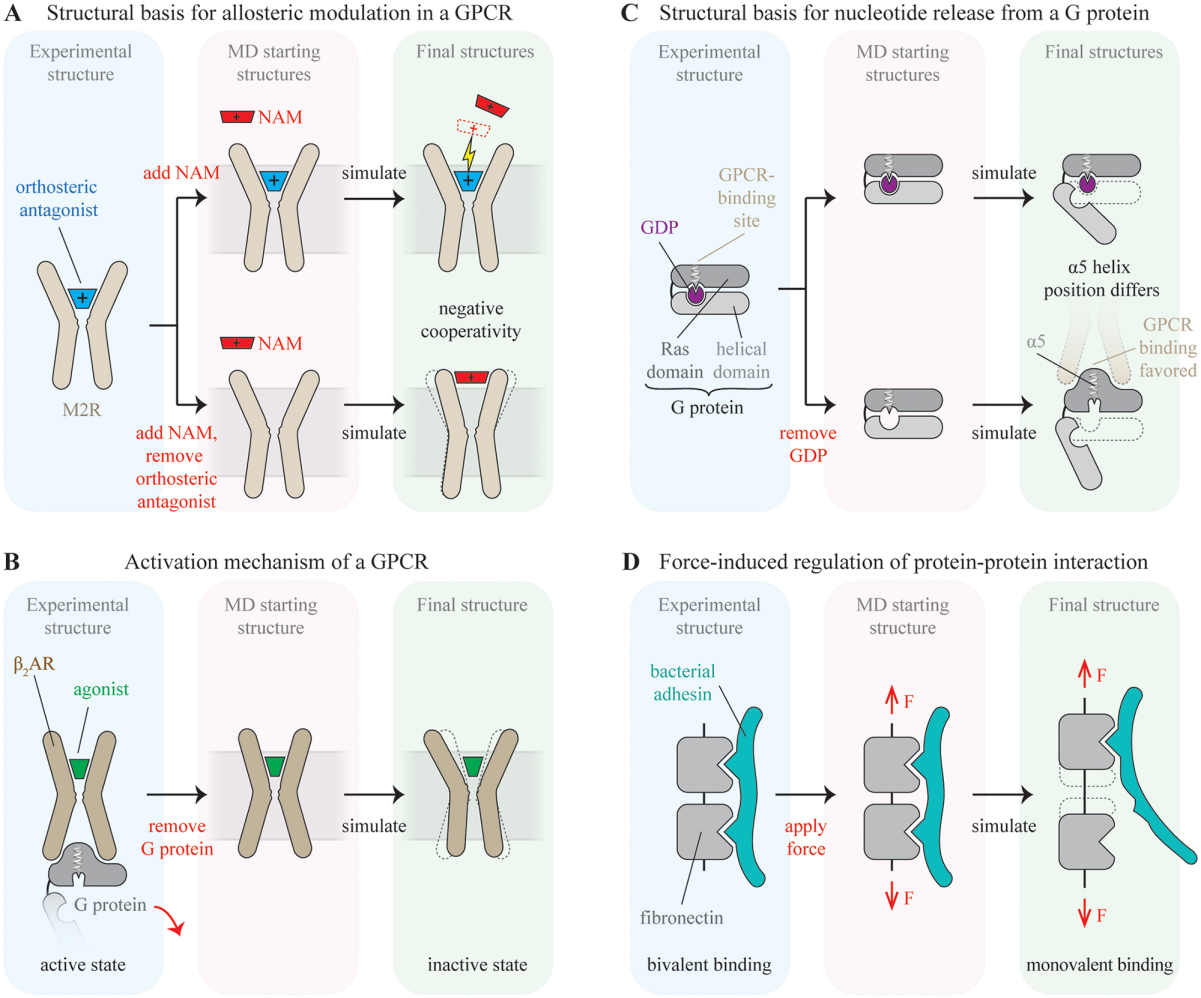
Simulation schematics for case studies. Key perturbations to the experimental structure are indicated in red text. (A) Binding and cooperativity of a negative allosteric modulator (NAM) in the M2 muscarinic receptor (M2R). The binding pose of the NAM and its cooperativity with the orthosteric ligand are probed by performing unguided binding simulations with an unliganded receptor and a receptor with bound orthosteric ligand. Negative cooperativity leads to stronger binding of the NAM to the unliganded M2R. Simulations indicate that cooperativity in this system is due both to direct electrostatic repulsion between cationic ligands and coupled conformational changes of the two binding sites. (B) Activation mechanism of the β_2_-adrenergic receptor (β_2_AR). Removal of the bound G protein from the active-state, agonist-bound crystal structure of β_2_AR leads to a spontaneous transition to the inactive state in simulation, capturing the activation process in reverse. (C) Mechanism of GPCR-catalyzed nucleotide release from a heterotrimeric G protein. Simulations of a G protein with and without bound guanosine diphosphate (GDP) suggested that nucleotide release from a G protein—which leads to G protein activation—takes place via a previously unexpected mechanism. (D) Force-induced uncoupling of a bacterial adhesin from a fibronectin fragment. MD simulations of a fibronectin–adhesin complex led to the discovery that application of stretching forces to fibronectin reduces its affinity to adhesin.

In our first sample study, we investigated the binding modes and molecular mechanisms of allosteric modulators of a G protein-coupled receptor (GPCR) (Fig 2A) [17]. Approximately one-third of all drugs act by binding to GPCRs, generally at the same site as the native ligands of these receptors—a site known as the orthosteric site. There is a great deal of pharmaceutical interest, however, in developing allosteric modulators, which bind at other sites on GPCRs and modulate the affinity of orthosteric ligands [57,58]. A number of allosteric modulators have been identified experimentally, but before we performed this study, it was not clear how they bound to their receptors or why they affected ligand affinity at the orthosteric site.

We worked with the M2 muscarinic acetylcholine receptor (M2R), a prototypical GPCR for which a substantial number of allosteric modulators have been identified experimentally and for which a crystal structure was available [59]. Because the crystal structure had an antagonist bound in the orthosteric site, we focused on several allosteric modulators known to affect the binding affinity of orthosteric antagonists, particularly the widely used antagonist N-methyl scopolamine (NMS).

To identify the binding sites and poses of the allosteric modulators, we performed a set of MD simulations in which each modulator was allowed to diffuse around the receptor until it bound spontaneously. Repeated binding simulations with several modulators showed not only that the ligands consistently bound an extracellular-facing vestibule on the receptor but also that the ligand binding mode was governed in all cases by a set of cation-π interactions between protein and ligand, rather than by previously proposed interactions involving electrostatic attraction or aromatic–aromatic contacts. To confirm our predicted ligand binding poses, we computationally designed a set of M2R mutants intended to increase or decrease allosteric modulator affinity. The affinities of two allosteric modulators for each of these mutants were then determined through radioligand binding experiments. In every case, the affinity changed in the direction predicted.

Next, we sought to determine the mechanism by which the allosteric modulators affect the affinity of the orthosteric ligand NMS. Our study included both modulators that decrease the affinity of the orthosteric ligand, known as negative allosteric modulators (NAMs), and modulators that increase the affinity of the orthosteric ligand, known as positive allosteric modulators (PAMs).

The bidirectional symmetry of allosteric systems dictates that a bound orthosteric ligand will also affect the binding of allosteric modulators: the orthosteric ligand will decrease the affinity of NAMs and increase the affinity of PAMs. We thus performed simulations of each allosteric modulator binding in both the presence and absence of the orthosteric ligand (in essence, the “perturbation” required to uncover the allosteric mechanism). Because the allosteric modulators bind and dissociate far more quickly than the orthosteric ligand, this was much more practical than performing simulations of orthosteric ligand binding in the presence and absence of allosteric modulators.

Analysis of these simulation results revealed two mechanisms of cooperativity between the allosteric modulators and the orthosteric ligand. The first, interestingly, did not require any structural change: electrostatic repulsion between charged allosteric and orthosteric ligands destabilized the binding of one in the presence of the other. The second mechanism did involve structural change: the orthosteric ligand increased the width of both the allosteric and orthosteric binding sites, and the PAMs had a similar effect; thus, the presence of either the orthosteric ligand or a PAM increased the affinity of the other.

We used our understanding of this mechanism to design an allosteric modulator with altered cooperativity: we added bulk to a modulator such that its binding was predicted computationally to require a wider geometry of the allosteric site. We then showed experimentally that this chemical modification shifted the modulator’s cooperativity with the orthosteric ligand in the positive direction. Our results may thus help guide the rational design of allosteric GPCR drugs.

Several studies have also used MD simulations to examine the allosteric effects of sodium ions at various GPCRs [60–62] and to show that various orthosteric ligands bind transiently at allosteric sites on their way to and from the orthosteric site [15, 63–65].

### Case Study 2: Mechanism of GPCR activation

We next turn to a study that investigates allostery between two distant sites on a protein, mediated by a conformational change of the entire protein (Fig 2B) [66]. Here, we sought to determine the mechanism of GPCR activation—the process by which binding of certain ligands (known as agonists) to the extracellular-facing orthosteric site favors a structural change that allows the intracellular side of the GPCR to bind to a G protein. This allosteric process underlies the basic function of GPCRs, which is to stimulate intracellular signaling when agonists are detected in the extracellular environment [67]. A number of other MD studies have addressed similar questions in a variety of GPCRs [68–72].

We aimed to discover how the agonist stabilizes the active state—that is, to identify the allosteric pathway connecting the orthosteric site to the intracellular G protein–coupling interface, as well as the nature of the coupling along that pathway. We focused on the β_2_-adrenergic receptor (β_2_AR), an archetypal GPCR for which crystal structures were available in both an inactive state (bound to various orthosteric ligands) [73] and an active state (bound simultaneously to an orthosteric agonist and a G protein or G protein–mimetic nanobody) [74,75].

A straightforward approach to studying the β_2_AR activation mechanism by MD would be to initiate simulations from the receptor’s inactive state, with an agonist placed in the orthosteric site. Unfortunately, the estimated timescales of receptor activation are many milliseconds, substantially beyond the timescales that were accessible to MD simulations.

We found, however, that simulations of an agonist-bound receptor initiated from the active state, with the G protein removed, transitioned spontaneously to the inactive state within a few microseconds. Indeed, a crystallographic structure of an agonist-bound β_2_AR with no intracellular binding partner was also inactive [76]; the intracellular binding partner plays a key role in stabilizing the fully active β_2_AR conformation.

Exploiting the principle of microscopic reversibility, we reasoned that simulations of receptor deactivation—the transition of an agonist-bound receptor from the active state to the inactive state—could be used to infer the mechanism of the reverse process, activation, in which an agonist-bound receptor transitions to the active state. We thus performed and analyzed several dozen such simulations and identified residues that acted as connectors between the extracellular-facing orthosteric site and the receptor’s intracellular face. Interestingly, structural changes to these different regions did not occur simultaneously or in a well-defined sequence. Instead, the various regions were loosely coupled, with each one more likely to assume an active-like conformation when its neighbors did so. We also found that the transition between the receptor’s active and inactive states generally proceeded through a well-defined, metastable intermediate state.

One caveat is that we did not simulate G protein dissociation, an event that would take place on far longer timescales, and our simulations may thus not reflect activation and deactivation at equilibrium. The receptor might follow a different pathway between inactive and active states in the presence of a G protein, particularly if such a binding partner is necessary to induce early conformational changes on the activation pathway. Experimental data suggests, however, that activation-like conformational fluctuations of the agonist-bound receptor take place even in the absence of an intracellular binding partner [74,77]. In addition, a subsequent nuclear magnetic resonance (NMR) study confirmed several of our computational predictions, including the loose nature of the allosteric coupling involved in activation and the existence of an intermediate state with properties similar to those suggested by our simulations [77].

### Case Study 3: Mechanism of nucleotide release in heterotrimeric G proteins

We next shift our focus to a study of allostery in a receptor–G protein complex, in which we asked how the interaction of a receptor (GPCR) with a heterotrimeric G protein accelerates GDP release from the G protein (Fig 2C) [78]. This process—which leads directly to nucleotide exchange and G protein activation—has long been viewed as a cornerstone of cell signaling, but its mechanism had remained unclear [79]. Simulations implicated a mechanism substantially different from those hypothesized previously.

The existence of crystal structures representing both a GDP-bound G protein [80] and a nucleotide free receptor–G protein complex [75], with distinct structural differences in the conformation of the G protein, made MD a particularly attractive method for studying the mechanistic basis for this process. The former crystal structure shows two domains, the Ras domain and the helical domain, pinched together around GDP. In the latter structure, the two domains appear to have swung apart in a hinge-like fashion. Based on this structural data, one might hypothesize that binding of a receptor to a G protein forces domain separation, thus enabling nucleotide release.

Surprisingly, when we initiated simulations from the closed, GDP-bound G protein structure, the helical and Ras domains separated spontaneously and frequently. This separation was more than sufficient to clear a pathway for GDP exit, but GDP remained bound. (Smaller interdomain motions had previously been observed in shorter MD simulations of G proteins [81–85].) The domains separated further in simulation when GDP was removed, but the hinge-like domain opening motion did not show any clear evidence of allosteric coupling to the receptor-binding interface of the G protein. These observations suggest that the receptor does not act on the G protein by increasing domain separation.

So what does the receptor do to accelerate nucleotide release from the G protein? Directly simulating the process of receptor–G protein association was not feasible, as it occurs on timescales longer than those we could access in simulation. By exploiting the principle of bidirectionality, however, we were able to address this question without directly simulating the association process.

In particular, physical principles imply that removal of GDP will favor G protein conformations that exhibit lower affinity for GDP over those that exhibit higher affinity for GDP. We thus compared simulations of a G protein with and without GDP bound, all initiated from the same structure solved in the absence of a receptor. We focused on the behavior of structural features on the G protein at the receptor-binding interface. We found that nucleotide removal favors the spontaneous transition of a Ras domain helix (α5) to a conformation seen in the receptor–G protein complex structure; this conformation presumably allows the G protein to couple more tightly to the receptor.

These simulations suggest that receptor binding causes a subtle conformational change within the Ras domain, reducing its affinity for GDP. This allows the bound GDP to escape when the domains separate spontaneously, in a manner that is independent of receptor binding. Simulations thus implicated a mechanism substantially different from those previously hypothesized on the basis of available experimental data.

Biophysical experiments, including several designed specifically to validate our computational results, supported this novel mechanism. Double electron–electron resonance spectroscopy experiments indicate that, in the presence of GDP, the distribution of distances between the helical domain and Ras domain has two distinct peaks, suggestive of an equilibrium between closed and open conformations [78,86]. To test the prediction that spontaneous domain separation plays an important role in nucleotide release, our collaborators engineered a G protein in which the Ras and helical domains are tethered together [78]. Kinetics experiments show that, in the absence of a GPCR, nucleotide release takes place about 20 times more slowly in this engineered G protein than in the wild type.

### Case Study 4: Mechanosensitive interaction between bacterial adhesins and fibronectin

Our final case study [87] differs from the previous ones in two ways (Fig 2D). First, the allosteric phenomenon itself was discovered by simulation before being verified experimentally. Second, the allostery involves regulation of a protein–protein interaction by a mechanical force rather than by binding of another ligand.

The large multidomain protein fibronectin plays important roles in cell adhesion, embryogenesis, and wound healing and exists in fibrillar form in the extracellular matrix of vertebrates [88]. Cells adhering to fibronectin fibers can generate mechanical forces that induce strain in individual fibronectin molecules [89], which convert tensile forces into biochemical cues [90]. Interestingly, several fibronectin domains are binding targets for pathogenic bacteria, which exploit the abundance of fibronectin in host tissue and have evolved fibronectin-binding adhesins to initiate host invasion [88].

Since fibronectin exhibits a wide range of stretched conformations in vivo, we wondered if the application of force to adhesin-bound fibrils altered fibronectin–adhesin interactions. If force applied to the ends of these fibers perturbed the distant adhesin–fibronectin binding interface, this would constitute a form of allostery. This study, then, aimed to discover whether an allosteric mechanism could govern a protein–protein interaction. Our goal was to investigate whether and how fibronectin domains that interact with bacterial adhesins undergo conformational changes in response to force and whether these putative changes lead to altered binding affinities between bacterial adhesins and fibronectin.

We attempted to answer these questions by simulating an NMR-resolved structure of a bacterial adhesin bound to a fibronectin fragment containing two domains [91], with stretching forces applied to the two ends of the fibronectin fragment. The forces used in these steered MD simulations were orders of magnitude greater than those observed biologically, allowing a force-based transition to take place on shorter, MD-accessible timescales. Nonetheless, over the course of our simulations, the fibronectin fragment stretched by an amount matching that observed in vivo as a result of cell-generated tension.

In our simulations, the fibronectin fragment transitioned into a stretched state in which the interdomain separation between the two fibronectin domains significantly increased. This was accompanied by partial unbinding of the bacterial adhesin: initially bound to both domains, the bacterial adhesin detached from one of the two domains in simulations but remained bound to the other. This transition is allosteric in nature because the forces applied at the two ends of the fibronectin fragment were distant from the binding motif from which the adhesin disengaged. In simulations of the same complex with no applied force, adhesin did not separate from fibronectin. Our conclusions from this computational analysis are in agreement with an independently conducted simulation study [92].

We used this data to make predictions about affinities within the fibronectin–adhesin complex: because bacterial adhesins bind to only a single domain when fibronectin is in a stretched state, reduction of the binding mode from bivalent to monovalent should result in lower affinities of bacterial adhesins for stretched fibronectin. Binding assays using fluorescently labeled bacterial adhesins and fibronectin fibers on stretchable silicone sheets subsequently confirmed that affinity of bacterial adhesins to fibronectin fibers varied with mechanical strain [87]. Furthermore, insertion of a few extra residues into the linker region of a bacterial adhesin abolished its mechanosensitive binding properties, because the engineered adhesin was able to maintain binding to two fibronectin domains even when their interdomain distance increased upon stretching [93].

Application of steered MD to probe force-induced allostery in other systems has demonstrated that results obtained through this technique agree well with experimental data: studied systems include integrins [94], large-conductance mechanosensitive channels [95], bacterial pili [96], and the focal adhesion proteins talin and vinculin [97,98].

## Conclusion

The conceptual approaches described here for studying allostery are general and powerful enough for application to a wide variety of biomolecular systems and problems. Yet much remains to be done. We lack a simple “recipe” for determining mechanisms of allostery computationally. We also need better approaches for mining large quantities of simulation data, particularly in cases in which the changes involved are subtle. As analysis methods continue to improve and as MD simulations become faster and more accurate, we envision that insights from simulation will play an ever greater role in discovering the structural basis for allostery, designing allosteric drugs, and engineering allosteric proteins.

## References

[1] Changeux J-P, Edelstein SJ. Allosteric mechanisms of signal transduction. Science. 2005;308:1424–8. 15933191 10.1126/science.1108595

[2] Motlagh HN, Wrabl JO, Li J, Hilser VJ. The ensemble nature of allostery. Nature. 2014;508:331–9. 10.1038/nature13001 24740064 PMC4224315

[3] Nussinov R, Tsai C-J. Allostery in disease and in drug discovery. Cell. 2013;153:293–305. 10.1016/j.cell.2013.03.034 23582321

[4] Raman S, Taylor N, Genuth N, Fields S, Church GM. Engineering allostery. Trends in Genetics. 2014;30:521–8. 10.1016/j.tig.2014.09.004 25306102 PMC4254034

[5] Cui Q, Karplus M. Allostery and cooperativity revisited. Protein Science. 2008;17:1295–307. 10.1110/ps.03259908 18560010 PMC2492820

[6] Hilser VJ, Wrabl JO, Motlagh HN. Structural and energetic basis of allostery. Annual Review of Biophysics. 2012;41:585–609. 10.1146/annurev-biophys-050511-102319 22577828 PMC3935618

[7] Tsai CJ, Nussinov R. A unified view of "how allostery works". PLoS Comput Biol. 2014;10 10.1371/journal.pcbi.1003394. 24516370 PMC3916236

[8] Swain JF, Gierasch LM. The changing landscape of protein allostery. Current Opinion in Structural Biology. 2006;16:102–8. 16423525 10.1016/j.sbi.2006.01.003

[9] Karplus M, Kuriyan J. Molecular dynamics and protein function. Proceedings of the National Academy of Sciences. 2005;102:6679–85. . 15870208 10.1073/pnas.0408930102 PMC1100762

[10] Dror RO, Dirks RM, Grossman JP, Xu H, Shaw DE. Biomolecular simulation: A computational microscope for molecular biology. Annual Review of Biophysics. 2012;41:429–52. 10.1146/annurev-biophys-042910-155245 22577825

[11] Collier G, Ortiz V. Emerging computational approaches for the study of protein allostery. Archives of Biochemistry and Biophysics. 2013;538:6–15. 10.1016/j.abb.2013.07.025 23933229

[12] Elber R. Simulations of allosteric transitions. Current Opinion in Structural Biology. 2011;21:167–72. 10.1016/j.sbi.2011.01.012 21333527

[13] Feher VA, Durrant JD, Van Wart AT, Amaro RE. Computational approaches to mapping allosteric pathways. Current Opinion in Structural Biology. 2014;25:98–103. 10.1016/j.sbi.2014.02.004 24667124 PMC4040315

[14] Buch I, Giorgino T, De Fabritiis G. Complete reconstruction of an enzyme-inhibitor binding process by molecular dynamics simulations. Proceedings of the National Academy of Sciences. 2011;108:10184–9. 10.1073/pnas.1103547108. . 21646537 PMC3121846

[15] Dror RO, Pan AC, Arlow DH, Borhani DW, Maragakis P, Shan Y, et al Pathway and mechanism of drug binding to G-protein-coupled receptors. Proceedings of the National Academy of Sciences. 2011;108:13118–23. 10.1073/pnas.1104614108. . 21778406 PMC3156183

[16] Shan Y, Kim ET, Eastwood MP, Dror RO, Seeliger MA, Shaw DE. How does a drug molecule find its target binding site? Journal of the American Chemical Society. 2011;133:9181–3. 10.1021/ja202726y 21545110 PMC3221467

[17] Dror RO, Green HF, Valant C, Borhani DW, Valcourt JR, Pan AC, et al Structural basis for modulation of a G-protein-coupled receptor by allosteric drugs. Nature. 2013;503:295–9. 10.1038/nature12595 24121438

[18] Doerr S, De Fabritiis G. On-the-fly learning and sampling of ligand binding by high-throughput molecular simulations. Journal of Chemical Theory and Computation. 2014;10:2064–9. 10.1021/ct400919u 26580533

[19] Durrant JD, McCammon JA. Molecular dynamics simulations and drug discovery. BMC Biology. 2011;9:71 10.1186/1741-7007-9-71 22035460 PMC3203851

[20] Nichols SE, Swift RV, Amaro RE. Rational prediction with molecular dynamics for hit identification. Current Topics in Medicinal Chemistry. 2012;12:2002–12. 23110535 10.2174/156802612804910313 PMC3636520

[21] Di Pizio A, Niv MY. Computational studies of smell and taste receptors. Israel Journal of Chemistry. 2014;54:1205–18. http://onlinelibrary.wiley.com/doi/10.1002/ijch.201400027/abstract 10.1002/ijch.201400027

[22] Wassman CD, Baronio R, Demir Ö, Wallentine BD, Chen C-K, Hall LV, et al Computational identification of a transiently open L1/S3 pocket for reactivation of mutant p53. Nature Communications. 2013;4:1407 10.1038/ncomms2361 23360998 PMC3562459

[23] Bakan A, Nevins N, Lakdawala AS, Bahar I. Simulations in the presence of probe molecules. Journal of Chemical Theory and Computation. 2012;8(7):2435–2447. 22798729 10.1021/ct300117j PMC3392909

[24] Bowman GR, Bolin ER, Hart KM, Maguire BC, Marqusee S. Discovery of multiple hidden allosteric sites by combining Markov state models and experiments. Proceedings of the National Academy of Sciences. 2015;112(9):2734–9. 10.1073/pnas.1417811112 PMC4352775 25730859

[25] Ivetac A, Andrew McCammon J. Mapping the druggable allosteric space of G-protein coupled receptors: A fragment-based molecular dynamics approach. Chemical Biology and Drug Design. 2010;76:201–17. 10.1111/j.1747-0285.2010.01012.x 20626410 PMC2918726

[26] Tan YS, Śledź P, Lang S, Stubbs CJ, Spring DR, Abell C, et al Using ligand-mapping simulations to design a ligand selectively targeting a cryptic surface pocket of polo-like kinase 1. Angewandte Chemie—International Edition. 2012;51:10078–81. 10.1002/anie.201205676 . 22961729 PMC3547296

[27] Humphrey W, Dalke A, Schulten K. VMD: Visual molecular dynamics. Journal of Molecular Graphics. 1996;14:33–8. 8744570 10.1016/0263-7855(96)00018-5

[28] LeVine MV, Weinstein H. NbIT—A New information theory-based analysis of allosteric mechanisms reveals residues that underlie function in the leucine transporter LeuT. PLoS Comput Biol. 2014;10(5):e1003603 10.1371/journal.pcbi.1003603 24785005 PMC4006702

[29] McClendon CL, Friedland G, Mobley DL, Amirkhani H, Jacobson MP. Quantifying correlations between allosteric sites in thermodynamic ensembles. Journal of Chemical Theory and Computation. 2009;5:2486–502. 20161451 10.1021/ct9001812 PMC2790287

[30] Kong Y, Karplus M. The signaling pathway of rhodopsin. Structure. 2007;15:611–23. 17502106 10.1016/j.str.2007.04.002

[31] Sethi A, Eargle J, Black AA, Luthey-Schulten Z. Dynamical networks in tRNA:protein complexes. Proceedings of the National Academy of Sciences. 2009;106:6620–5. 10.1073/pnas.0810961106. 19351898 PMC2672494

[32] Van Wart AT, Durrant J, Votapka L, Amaro RE. Weighted implementation of suboptimal paths (WISP): An optimized algorithm and tool for dynamical network analysis. Journal of Chemical Theory and Computation. 2014;10:511–7. 24803851 10.1021/ct4008603 PMC3958135

[33] Van Wart AT, Eargle J, Luthey-Schulten Z, Amaro RE. Exploring residue component contributions to dynamical network models of allostery. Journal of Chemical Theory and Computation. 2012;8:2949–61. 23139645 10.1021/ct300377a PMC3489502

[34] Weinkam P, Pons J, Sali A. Structure-based model of allostery predicts coupling between distant sites. Proceedings of the National Academy of Sciences. 2012;109:4875–80. 10.1073/pnas.1116274109. . 22403063 PMC3324024

[35] Fan Z, Dror RO, Mildorf TJ, Piana S, Shaw DE. Identifying localized changes in large systems: Change-point detection for biomolecular simulations. Proceedings of the National Academy of Sciences. 2015;112:201415846 10.1073/pnas.1415846112. . 26025225 PMC4475967

[36] Shukla D, Hernandez CX, Weber JK, Pande VS. Markov state models provide insights into dynamic modulation of protein function. Accounts of Chemical Research. 2015;48(2):414–22. 10.1021/ar5002999 25625937 PMC4333613

[37] Yang S, Banavali NK, Roux B. Mapping the conformational transition in Src activation by cumulating the information from multiple molecular dynamics trajectories. Proceedings of the National Academy of Sciences. 2009;106:3776–81. 10.1073/pnas.0808261106. . 19225111 PMC2656156

[38] Malmstrom RD, Kornev AP, Taylor SS, Amaro RE. Allostery through the computational microscope: cAMP activation of a canonical signalling domain. Nature Communications. 2015;6:7588 10.1038/ncomms8588 26145448 PMC4504738

[39] Vanatta DK, Shukla D, Lawrenz M, Pande VS. A network of molecular switches controls the activation of the two-component response regulator NtrC. Nature Communications. 2015;6:7283 10.1038/ncomms8283 26073186

[40] Seifert C, Gräter F. Protein mechanics: How force regulates molecular function. Biochimica et Biophysica Acta. 2013;1830:4762–8. 10.1016/j.bbagen.2013.06.005 23791949

[41] Jensen MØ, Borhani DW, Lindorff-Larsen K, Maragakis P, Jogini V, Eastwood MP, et al Principles of conduction and hydrophobic gating in K^+^ channels. Proceedings of the National Academy of Sciences. 2010;107:5833–8. 10.1073/pnas.0911691107. 20231479 PMC2851896

[42] Foda ZH, Shan Y, Kim ET, Shaw DE, Seeliger MA. A dynamically coupled allosteric network underlies binding cooperativity in Src kinase. Nature Communications. 2015;5:1–10. 10.1038/ncomms6939. . 25600932 PMC4300553

[43] Isralewitz B, Gao M, Schulten K. Steered molecular dynamics and mechanical functions of proteins. Current Opinion in Structural Biology. 2001;11:224–30. 11297932 10.1016/s0959-440x(00)00194-9

[44] Paci E, Karplus M. Forced unfolding of fibronectin type 3 modules: an analysis by biased molecular dynamics simulations. Journal of Molecular Biology. 1999;288:441–59. 10329153 10.1006/jmbi.1999.2670

[45] Ovchinnikov V, Karplus M. Analysis and elimination of a bias in targeted molecular dynamics simulations of conformational transitions: Application to calmodulin. Journal of Physical Chemistry. 2012;116:8584–603. 10.1021/jp212634z. . 22409258 PMC3406239

[46] Atilgan C, Atilgan AR. Perturbation-response scanning reveals ligand entry-exit mechanisms of ferric binding protein. PLoS Comput Biol. 2009;5(10):e1000544 10.1371/journal.pcbi.1000544 19851447 PMC2758672

[47] Gerek ZN, Ozkan SB. Change in allosteric network affects binding affinities of PDZ domains: Analysis through perturbation response scanning. PLoS Comput Biol. 2011;7:18–25. 10.1371/journal.pcbi.1002154. 21998559 PMC3188487

[48] Sharp K, Skinner JJ. Pump-probe molecular dynamics as a tool for studying protein motion and long range coupling. Proteins. 2006;65:347–61. 16933296 10.1002/prot.21146

[49] Likic VA, Gooley PR, Speed TP, Strehler EE. A statistical approach to the interpretation of molecular dynamics simulations of calmodulin equilibrium dynamics. Protein Science 2005;14(12):2955–63. 16322577 10.1110/ps.051681605 PMC2253239

[50] Astumian RD. Microscopic reversibility as the organizing principle of molecular machines. Nature Nanotechnology. 2012;7(11):684–8. 10.1038/nnano.2012.188 23132220

[51] Lewis GN. A new principle of equilibrium. Proceedings of the National Academy of Sciences. 1925;11:179–83. 10.1073/pnas.11.3.179 PMC1085913 16576866

[52] Bhatt D, Zuckerman DM. Beyond microscopic reversibility: Are observable nonequilibrium processes precisely reversible? Journal of Chemical Theory and Computation. 2011;7(8):2520–7. 21869866 10.1021/ct200086k PMC3159166

[53] Lindorff-Larsen K, Maragakis P, Piana S, Eastwood MP, Dror RO, Shaw DE. Systematic validation of protein force fields against experimental data. PLoS ONE. 2012;7:1–6. 10.1371/annotation/8301b5d4-1ba3-40e7-8fcd-3e169b967044. 22384157 PMC3285199

[54] Dror RO, Jensen MØ, Borhani DW, Shaw DE. Exploring atomic resolution physiology on a femtosecond to millisecond timescale using molecular dynamics simulations. The Journal of General Physiology. 2010;135:555–62. 10.1085/jgp.200910373 20513757 PMC2888062

[55] Harvey MJ, De Fabritiis G. High-throughput molecular dynamics: The powerful new tool for drug discovery. Drug Discovery Today. 2012;17:1059–62. 10.1016/j.drudis.2012.03.017 22504137

[56] Schwantes CR, McGibbon RT, Pande VS. Perspective: Markov models for long-timescale biomolecular dynamics. The Journal of Chemical Physics. 2014;141:090901 10.1063/1.4895044 25194354 PMC4156582

[57] Christopoulos A. Advances in GPCR allostery: from function to structure. Molecular Pharmacology. 2014:463–78. 10.1124/mol.114.094342 25061106

[58] Conn PJ, Christopoulos A, Lindsley CW. Allosteric modulators of GPCRs: a novel approach for the treatment of CNS disorders. Nature Reviews Drug discovery. 2009;8:41–54. 10.1038/nrd2760 19116626 PMC2907734

[59] Haga K, Kruse AC, Asada H, Yurugi-Kobayashi T, Shiroishi M, Zhang C, et al Structure of the human M2 muscarinic acetylcholine receptor bound to an antagonist. Nature. 2012;482:547–51. 10.1038/nature10753 22278061 PMC3345277

[60] Gutiérrez-De-Terán H, Massink A, Rodríguez D, Liu W, Han GW, Joseph JS, et al The role of a sodium ion binding site in the allosteric modulation of the A2A adenosine G protein-coupled receptor. Structure. 2013;21:2175–85. 10.1016/j.str.2013.09.020 24210756 PMC3858454

[61] Miao Y, Caliman Alisha D, McCammon JA. Allosteric effects of sodium ion binding on activation of the M3 muscarinic G-protein-coupled receptor. Biophysical Journal. 2015;108:1796–806. 10.1016/j.bpj.2015.03.003 25863070 PMC4390834

[62] Selent J, Sanz F, Pastor M, De Fabritiis G. Induced effects of sodium ions on dopaminergic G-protein coupled receptors. PLoS Comput Biol. 2010;6(8):e1000884 10.1371/journal.pcbi.1000884 20711351 PMC2920834

[63] Dror RO, Pan AC, Arlow DH, Shaw DE. Probing the conformational dynamics of GPCRs with molecular dynamics simulation. G Protein-Coupled Receptors: From Structure to Function. 2011;(8):384–400.

[64] Kappel K, Miao Y, McCammon JA. Accelerated molecular dynamics simulations of ligand binding to a muscarinic G-protein-coupled receptor. Quarterly Reviews of Biophysics. 2015;48(04):479–87. 10.1017/S0033583515000153. . 26537408 PMC5435230

[65] Kruse AC, Hu J, Pan AC, Arlow DH, Rosenbaum DM, Rosemond E, et al Structure and dynamics of the M3 muscarinic acetylcholine receptor. Nature. 2012;482:552–6. 10.1038/nature10867 22358844 PMC3529910

[66] Dror RO, Arlow DH, Maragakis P, Mildorf TJ, Pan AC, Xu H, et al Activation mechanism of the ß2-adrenergic receptor. Proceedings of the National Academy of Sciences. 2011;108:18684–9. 10.1073/pnas.1110499108. . 22031696 PMC3219117

[67] Katritch V, Cherezov V, Stevens RC. Structure-function of the G protein-coupled receptor superfamily. Annual Review of Pharmacology and Toxicology. 2013;53:531–56. 10.1146/annurev-pharmtox-032112-135923 23140243 PMC3540149

[68] Bhattacharya S, Vaidehi N. Computational mapping of the conformational transitions in agonist selective pathways of a G-protein coupled receptor. Journal of the American Chemical Society. 2010;132(14):5205–14. 10.1021/ja910700y 20235532

[69] Kohlhoff KJ, Shukla D, Lawrenz M, Bowman GR, Konerding DE, Belov D, et al Cloud-based simulations on Google Exacycle reveal ligand modulation of GPCR activation pathways. Nature Chemistry. 2014;6:15–21. 10.1038/nchem.1821 24345941 PMC3923464

[70] Lee Y, Choi S, Hyeon C. Communication over the network of binary switches regulates the activation of A2A adenosine receptor. PLOS Comput Biol. 2015;11:e1004044 10.1371/journal.pcbi.1004044 25664580 PMC4322061

[71] Vanni S, Rothlisberger U. A closer look into G protein coupled receptor activation: X-ray crystallography and long-scale molecular dynamics simulations. Current Medicinal Chemistry. 2012;19(8):1135–45. 22300050 10.2174/092986712799320493

[72] Yuan S, Filipek S, Palczewski K, Vogel H. Activation of G-protein-coupled receptors correlates with the formation of a continuous internal water pathway. Nature Communications. 2014;5:4733 10.1038/ncomms5733 25203160

[73] Rosenbaum DM, Cherezov V, Hanson MA, Rasmussen SGF, Thian FS, Kobilka TS, et al GPCR engineering yields high-resolution structural insights into b2-adrenergic receptor function. Science. 2007;318:1266–73. 17962519 10.1126/science.1150609

[74] Rasmussen SGF, Choi H-j, Fung JJ, Pardon E, Chae PS, DeVree BT, et al Structure of a nanobody-stabilized active state of the β2 adrenoceptor. Nature. 2011;469:175–80. 10.1038/nature09648 21228869 PMC3058308

[75] Rasmussen SGF, DeVree BT, Zou Y, Kruse AC, Chung KY, Kobilka TS, et al Crystal structure of the β2 adrenergic receptor–Gs protein complex. Nature. 2011;477:549–55. 10.1038/nature10361 21772288 PMC3184188

[76] Rosenbaum DM, Zhang C, Lyons JA, Holl R, Aragao D, Arlow DH, et al Structure and function of an irreversible agonist-beta(2) adrenoceptor complex. Nature. 2011;469(7329):236–40. 10.1038/nature09665 21228876 PMC3074335

[77] Nygaard R, Zou Y, Dror RO, Mildorf TJ, Arlow DH, Manglik A, et al The dynamic process of β2-adrenergic receptor activation. Cell. 2013;152:532–42. 10.1016/j.cell.2013.01.008 23374348 PMC3586676

[78] Dror RO, Mildorf TJ, Hilger D, Manglik A, Borhani DW, Arlow DH, et al Structural basis for nucleotide exchange in heterotrimeric G proteins. Science. 2015;348:1361–5. 10.1126/science.aaa5264 26089515 PMC4968074

[79] Oldham WM, Hamm HE. Heterotrimeric G protein activation by G-protein-coupled receptors. Nature Reviews Molecular Cell Biology. 2008;9:60–71. 18043707 10.1038/nrm2299

[80] Wall MA, Coleman DE, Lee E, Iñiguez-Lluhi JA, Posner BA, Gilman AG, et al The structure of the G protein heterotrimer Gi alpha 1 beta 1 gamma 2. Cell. 1995;83:1047–58. 8521505 10.1016/0092-8674(95)90220-1

[81] Ceruso MA, Periole X, Weinstein H. Molecular dynamics simulations of transducin: interdomain and front to back communication in activation and nucleotide exchange. Journal of Molecular Biology. 2004;338:469–81. 15081806 10.1016/j.jmb.2004.02.064

[82] Jones JC, Jones AM, Temple BRS, Dohlman HG. Differences in intradomain and interdomain motion confer distinct activation properties to structurally similar G proteins. Proceedings of the National Academy of Sciences. 2012;109:7275–9. 10.1073/pnas.1202943109. . PMC3358857 22529365

[83] Khafizov K, Lattanzi G, Carloni P. G protein inactive and active forms investigated by simulation methods. Proteins: Structure, Function and Bioinformatics. 2009;75:919–30. 10.1002/prot.22303. . 19089952

[84] Louet M, Perahia D, Martinez J, Floquet N. A concerted mechanism for opening the GDP binding pocket and release of the nucleotide in hetero-trimeric G-proteins. Jounal of Molecular Biology. 2011;411:298–312. 10.1016/j.jmb.2011.05.034. . 21663745

[85] Mello LV, van Aalten DM, Findlay JB. Dynamic properties of the guanine nucleotide binding protein alpha subunit and comparison of its guanosine triphosphate hydrolase domain with that of ras p21. Biochemistry. 1998;37(9):3137–42. 9485466 10.1021/bi971402v

[86] Van Eps N, Preininger AM, Alexander N, Kaya AI, Meier S, Meiler J, et al Interaction of a G protein with an activated receptor opens the interdomain interface in the alpha subunit. Proceedings of the National Academy of Sciences. 2011;108(23):9420–4. 10.1073/pnas.1105810108. . 21606326 PMC3111277

[87] Chabria M, Hertig S, Smith ML, Vogel V. Stretching fibronectin fibres disrupts binding of bacterial adhesins by physically destroying an epitope. Nature Communications. 2010;1:135 10.1038/ncomms1135 21139580 PMC3105298

[88] Henderson B, Nair S, Pallas J, Williams MA. Fibronectin: a multidomain host adhesin targeted by bacterial fibronectin-binding proteins. FEMS Microbiology Reviews. 2010:1–54. 10.1111/j.1574-6976.2010.00243.x. 20695902

[89] Baneyx G, Baugh L, Vogel V. Fibronectin extension and unfolding within cell matrix fibrils controlled by cytoskeletal tension. Proceedings of the National Academy of Sciences. 2002;99:5139–43. . 11959962 10.1073/pnas.072650799 PMC122735

[90] Vogel V. Mechanotransduction involving multimodular proteins: converting force into biochemical signals. Annual Review of Biophysics and Biomolecular Structure. 2006;35:459–88. 16689645 10.1146/annurev.biophys.35.040405.102013

[91] Schwarz-Linek U, Werner JM, Pickford AR, Gurusiddappa S, Kim JH, Pilka ES, et al Pathogenic bacteria attach to human fibronectin through a tandem beta-zipper. Nature. 2003;423:177–81. 12736686 10.1038/nature01589

[92] Diao J, Maniotis AJ, Folberg R, Tajkhorshid E. Interplay of mechanical and binding properties of fibronectin type I. Theoretical Chemistry Accounts. 2010;125:397–405. 20824113 10.1007/s00214-009-0677-y PMC2932639

[93] Hertig S, Chabria M, Vogel V. Engineering mechanosensitive multivalent receptor-ligand interactions: Why the nanolinker regions of bacterial adhesins matter. Nano Letters. 2012;12:5162–8. 10.1021/nl302153h 22938173

[94] Jin M, Andricioaei I, Springer TA. Conversion between three conformational states of integrin I domains with a C-terminal pull spring studied with molecular dynamics. Structure. 2004;12:2137–47. 15576028 10.1016/j.str.2004.10.005

[95] Gullingsrud J, Schulten K. Gating of MscL studied by steered molecular dynamics. Biophysical Journal. 2003;85:2087–99. 14507677 10.1016/s0006-3495(03)74637-2 PMC1303438

[96] Baker JL, Biais N, Tama F. Steered molecular dynamics simulations of a type IV pilus probe initial stages of a force-induced conformational transition. PLoS Comput Biol. 2013;9:e1003032 10.1371/journal.pcbi.1003032 23592974 PMC3623709

[97] Del Rio A, Perez-Jimenez R, Liu R, Roca-Cusachs P, Fernandez JM, Sheetz MP. Stretching single talin rod molecules activates vinculin binding. Science. 2009;323:638–41. 10.1126/science.1162912 19179532 PMC9339221

[98] Hytönen VP, Vogel V. How force might activate talin’ s vinculin binding sites: SMD reveals a structural mechanism. PLoS Comput Biol. 2008;4(2):e24 10.1371/journal.pcbi.0040024 18282082 PMC2242828

